# Meta-transcriptomic comparison of two sponge holobionts feeding on coral- and macroalgal-dissolved organic matter

**DOI:** 10.1186/s12864-022-08893-y

**Published:** 2022-09-29

**Authors:** Sara Campana, Ana Riesgo, Evelien Jongepier, Janina Fuss, Gerard Muyzer, Jasper M. de Goeij

**Affiliations:** 1 Department of Freshwater and Marine Ecology, Institute for Biodiversity and Ecosystem Dynamics, University of Amsterdam, Post Office Box 94240, 1090 Amsterdam, GE Netherlands; 2 Department of Biodiversity and Evolutionary Biology, Museo Nacional de Ciencias Naturales (CSIC), Calle José Gutiérrez Abascal 2, 28006 Madrid, Spain; 3 Institute of Clinical Molecular Biology, Kiel University and University Medical Center Schleswig-Holstein, 24105 Kiel, Germany; 4 CARMABI Foundation, Piscaderabaai z/n, P.O. Box 2090, Willemstad, Curaçao

**Keywords:** Porifera, Sponge holobiont, Microbiota, RNA sequencing, meta-transcriptomics, Dissolved organic matter (DOM), Coral-DOM, Macroalgal-DOM

## Abstract

**Background:**

Sponge holobionts (i.e., the host and its associated microbiota) play a key role in the cycling of dissolved organic matter (DOM) in marine ecosystems. On coral reefs, an ecological shift from coral-dominated to algal-dominated ecosystems is currently occurring. Given that benthic corals and macroalgae release different types of DOM, in different abundances and with different bioavailability to sponge holobionts, it is important to understand how the metabolic activity of the host and associated microbiota change in response to the exposure to both DOM sources. Here, we look at the differential gene expression of two sponge holobionts 6 hours after feeding on naturally sourced coral- and macroalgal-DOM using RNA sequencing and meta-transcriptomic analysis.

**Results:**

We found a slight, but significant differential gene expression in the comparison between the coral- and macroalgal-DOM treatments in both the high microbial abundance sponge *Plakortis angulospiculatus* and the low microbial abundance sponge *Haliclona vansoesti*. In the hosts, processes that regulate immune response, signal transduction, and metabolic pathways related to cell proliferation were elicited. In the associated microbiota carbohydrate metabolism was upregulated in both treatments, but coral-DOM induced further lipid and amino acids biosynthesis, while macroalgal-DOM caused a stress response. These differences could be driven by the presence of distinct organic macronutrients in the two DOM sources and of small pathogens or bacterial virulence factors in the macroalgal-DOM.

**Conclusions:**

This work provides two new sponge meta-transcriptomes and a database of putative genes and genetic pathways that are involved in the differential processing of coral- versus macroalgal-DOM as food source to sponges with high and low abundances of associated microbes. These pathways include carbohydrate metabolism, signaling pathways, and immune responses. However, the differences in the meta-transcriptomic responses of the sponge holobionts after 6 hours of feeding on the two DOM sources were small. Longer-term responses to both DOM sources should be assessed to evaluate how the metabolism and the ecological function of sponges will be affected when reefs shift from coral towards algal dominance.

**Supplementary Information:**

The online version contains supplementary material available at 10.1186/s12864-022-08893-y.

## Background

Sponges and their associated microbiota (holobionts) are key marine ecosystem drivers because of their efficient uptake, processing, and release of organic and inorganic nutrients within their ecosystems [1–3]. An important food source used by sponges is dissolved organic matter (DOM) [4–8]. In the oceans, DOM is the main pool of organic matter and it is released by primary producers, such as phytoplankton, macroalgae, and corals [9–11]. In the past twenty years, considerable changes in benthic communities, such as a shift from coral to algal dominance, have occurred on many coral reefs as a consequence of climatic events in combination with direct anthropogenic disturbances [12–14]. Benthic algae, including turf algae and macroalgae, are found to release higher amounts of bioavailable DOM than corals [15, 16], which results in higher growth rates of ambient bacterioplankton, including the growth of pathogenic bacteria [16–19]. Recently, differential processing of naturally sourced coral- and macroalgal-DOM has been observed also in sponges, showing that macroalgal-DOM is generally more bioavailable to the sponge holobiont than coral-DOM [20, 21].

When exposed to coral- versus algal-DOM, seawater bacterioplankton communities not only shift in composition, but also adopt different metabolic strategies [16, 17]. After exposure to coral-DOM, bacterioplankton communities became dominated by Alphaproteobacteria, while Bacteroidetes, Gammaproteobacteria, and Cyanobacteria became more abundant after exposure to algal-DOM [17]. Furthermore, a metagenomic assessment of these bacterial groups showed a significant enrichment in genes encoding for the Embden–Meyerhof–Parnas (EMP) pathways on coral-dominated reefs, as opposed to the tricarboxylic acid (TCA) cycle, Entner-Doudoroff (ED), and pentose phosphate (PP) pathways on algal-dominated reefs [17]. Whereas the EMP pathway enriched on coral-dominated reefs is the most efficient in energy production, the direct breakdown of sugars in the ED, PP and TCA pathways rapidly remineralize the available organic carbon, but at a higher energetic cost [22, 23]. Some bacterial lineages present in the bacterioplankton are also found in association with sponges, but it is unknown whether similar, or any, shifts in sponge holobionts and their metabolic pathways are found when exposed to coral- versus macroalgal-DOM.

Metagenomic sequence analyses has provided insight into the putative metabolism of the sponge microbiota, revealing several microbial groups encoding genes for carbon, nitrogen, sulfur, and phosphate metabolism along with vitamin biosynthesis, as reviewed in [24–26]. A metagenomic study spanning seven sponge species and 25 microbial phyla suggested that some Chloroflexi, Poribacteria, Acidobacteria, Spirochaetes, and Latescibacteria are enriched in glycosyl hydrolases (GHs) acting on arabinose and fucose sugars, which are components of coral mucus (i.e., part of the coral-DOM pool) and macroalgal-DOM [27]. However, to confirm putative physiological responses and metabolic pathways employed by both host and associated microbiota and transcend ‘potential functions’ of sponges derived from metagenomics studies, it is required that molecular sequence data are coupled to hypothesis- driven experimental studies [28]. Recently, sponge-associated bacterial taxa of the sponge *Plakortis angulospiculatus* were shown to have an active metabolism in DOM processing by coupling DNA sequencing and stable isotope probing (DNA-SIP), for the first time in a marine holobiont [29]. Transcriptional responses to DOM-feeding by sponge host and its associated microbiota are at present not yet described.

In this study, we performed a meta-transcriptomic analysis on two sponge species—the high microbial abundance (HMA) species *Plakortis angulospiculatus* and the low microbial abundance (LMA) species *Haliclona vansoesti*—of which we previously assessed the processing (organic carbon/nitrogen assimilation and inorganic nutrient fluxes) of naturally sourced macroalgal- and coral-DOM [21]. Our previous study showed that in both sponge species there was up to two times higher assimilation of organic and inorganic nitrogen when sponges were fed with macroalgal- compared to coral-DOM. Here, we analysed the differential transcript expression of the sponge host and of the sponge-associated microbiota 6 hours after feeding on coral- and macroalgal-DOM and discuss how differential expression is linked with previously observed differences in coral- versus macroalgal-DOM processing.

## Methods

### Sample collection

As part of the study described in Campana et al. [21], individuals of the sponges *Plakortis angulospiculatus* [class Homoscleromorpha; HMA, encrusting 1–4 cm thick lobate/ficiform] and *Haliclona (Halichoclona) vansoesti* [class Demospongiae; LMA, encrusting 0.5–3 cm thick conulose] were collected on the fringing reef close to Piscadera Bay on Curaçao (12° 12′ N, 68° 96′ W), between 10 and 30 m water depth by SCUBA. After collection, sponges were trimmed to a size between 10 and 30 cm^2^ (leaving at least three functional oscula) and cleared of epibionts. Trimmed specimens were allowed to recover at the collection site for 3–4 weeks to ensure full recovery from collection and handling. Only visually healthy sponges (no tissue damage, open oscula as a measure for active pumping) were used in the experiments. The two sponge species were incubated with either coral- or macroalgal-DOM for 6 h, or diatom-DOM for 3 h (*n* = 3 per species x DOM source). Whereas the diatom-DOM source was artificially made in the laboratory, macroalgal- and coral-DOM were naturally sourced to better reflect the composition of DOM exudates released into the environment. All three DOM sources were enriched in ^13^C and ^15^N, using NaH^13^CO_3_ and Na^15^NO_3_, for tracing the assimilation of organic carbon and nitrogen by the sponges [21]. Briefly, the coral- and macroalgal-DOM were obtained by collection and filtration (0.7 μm) of the water containing the exudates released by the labeled corals or macroalgae, while diatom-DOM was obtained by lysis and filtration (0.2 μm) of the labeled diatom cells. The latter DOM source served as a highly labeled control to our naturally sourced macroalgae- and coral-DOM and was produced using axenic batch cultures of the cosmopolitan marine diatom *Phaeodactylum tricornutum.* For detailed description of the DOM sources production and labeling see Campana et al. [21]. Sponge individuals were transferred, without air exposure, to air-tight, stirred, incubation chambers, which were filled with coral-, macroalgal-, or diatom DOM. All incubations were conducted in the dark and dissolved oxygen concentration was monitored continuously with an optical probe (OXY-4, PreSens, Regensburg, Germany). Incubation chambers were placed in a flow-through aquarium to ensure near in situ temperature. At the end of the incubation, the sponge individuals were rinsed in non-labeled fresh seawater to remove excess tracer isotopes and dipped in Milli-Q water to remove salts before sampling the tissue for RNA. The sponge tissue samples included both pinacoderm and mesohyl and were snap frozen and stored in TRIzol® Reagent at − 80 °C until further processing.

### RNA extraction and sequencing

Total RNA was extracted from the sponge tissue samples using TRIzol® Reagent and the PureLink® RNA Mini Kit (Invitrogen) with on-column PureLink® DNase treatment, following the manufacturer’s protocol. The extracted total RNA was cleaned-up with the RNeasy MinElute Cleanup Kit (Qiagen), also following the manufacturer’s protocol. The final RNA concentrations were checked with the Qubit™ RNA BR Assay Kit and Qubit® 2.0 Fluorometer (Invitrogen, CA, USA). The RNA was stored at − 80 °C until further analysis. The quality of the extracted RNA was further assessed on a Qubit® 3.0 Fluorometer (Invitrogen, CA, USA) with the RNA BR Assay Kit and the TapeStation RNA ScreenTape at the Competence Centre for Genomic Analysis (CCGA) in Kiel, Germany, where the library preparation and sequencing took place. Ribosomal RNA from prokaryotes and eukaryotes was removed using the Illumina Ribo-Zero Plus rRNA depletion kit. Eighteen cDNA libraries (two species x three replicates x three DOM treatments) were then prepared with the TruSeq stranded total RNA kit according to the pre-release protocol and sequenced on one lane of the Illumina NovaSeq6000 S1 FlowCell, using a paired-end (150 bp length) sequencing strategy. The diatom-DOM treatment is included in our analysis to aid in the construction of complete reference transcriptomes (see below) for both species, but not in our differential expression analysis because we focused on the differences between the coral- and macroalgal-DOM, which were naturally sourced to better reflect the composition of DOM exudates released into the environment.

### Sequence quality control, transcriptome assembly, and annotation

Removal of adapter sequences and sequence quality was confirmed using the FastQC programme [30]. Low-quality regions of reads were trimmed using Trimmomatic v 0.39 [31] with the following settings: ILLUMINACLIP:./Adaptors.fa:2:30:10 LEADING:3 TRAILING:3 SLIDINGWINDOW: 4:28 MINLEN:36 where the Adaptors.fa file consisted of the specific Illumina indexes (oligonucleotide sequences) used for preparing the libraries. The resulting trimmed reads were then re-analysed with FastQC. To determine and eliminate ribosomal contamination, we used SortMeRNA v 4.2.0 [32]. The clean trimmed reads (three replicates x three DOM treatments) were then pooled separately for each species for the construction of a reference transcriptome using Trinity v 2.9.0 [33], with two non-standard settings: a minimum contig length of 200 bp and in silico read normalization.

The quality and completeness of the two reference transcriptomes were assessed on gVolante web server [34] using the Basic Universal Single Copy Orthologue (BUSCO) v5 [35] pipeline, selecting the eukaryotic, metazoan and bacteria BUSCO gene lists. For the annotation we performed two different analyses. First, we annotated the reference transcriptomes against the NCBI database *nr* using BLAST [36] and used MEGAN v 6.19.7 [37] for classifying the hits by kingdom. We then used DIAMOND v 2.0.6 [38] to refine our search using the more curated database of Swiss-Prot. Since taxon assignment in BLAST searches can sometimes be difficult for very conserved genes, we searched against two separate Swiss-Prot protein databases: a selection of all metazoan proteins and a selection of all prokaryotic proteins (cutoff *e*-value: 0.001). We then cross-checked both annotation files for more confident hit assignment, retaining all annotations for both databases for comparison. Further annotation was performed with Blast2GOPRO [39], to retrieve the Gene Ontology (GO) terms. Transdecoder v 5.5.0 (https://github.com/TransDecoder/TransDecoder) was used to identify putative coding regions within transcripts (ORFs ≥50 AA long). The KAAS-KEGG automatic annotation server v 2.1 [40] was used to gain an understanding of the recovery of complete pathways in our transcriptome. These were generated using the online tool rather than as integrated into Blast2GO, due to the increased functionality of the standalone server. The bi-directional best hit method was used to identify and annotate our contigs, with the protein sequences generated earlier used as the basis for these comparisons against a range of eukaryotic and prokaryotic species. KEGG pathways were reconstructed with the KEGG Mapper Reconstruct tool [41] based on K numbers identified from the KAAS-KEGG annotation. Gene mapping to KEGG pathways within the category ‘carbon metabolism’, ‘nitrogen metabolism’ and ‘ATP-binding cassette (ABC) transporters’ were compared in both sponge species.

### Differential expression and functional enrichment analyses

We assessed the differential transcript expression between the coral- and macroalgal-DOM feeding treatments in our two target sponges. First, alignment of the reads to the reference transcriptome and estimation of transcripts expression values were performed using RSEM [42] as packaged within the Trinity [33] module and Bowtie2 [43]. Then, the differential expression of transcripts between the two treatments was analysed using the Bioconductor package edgeR [44] within the Trinity [33] perl wrapper script, using the pairwise model (an exact test for the negative binomial distribution) with the following parameters: false discovery rate ≤ 0.0001 and minimum absolute (log2(a/b)) change of 2 (i.e., fourfold change), to minimize false positives. We excluded any differentially expressed transcripts where transcription was only detected in a single sample, prior to clustering and downstream analysis, to avoid spurious results caused by transient expression or contamination on single samples. Finally, the remaining differentially expressed transcripts were aligned with the GO terms and KEGG annotations tables. We performed the analyses using transcripts instead of genes to capture the most complete transcriptional response of the holobionts, given that a reference genome is not available for the species, and the patterns of alternative splicing are not yet known.

An additional GO enrichment analysis was conducted in the Blast2GOPRO platform [39]. We identified which functional GO categories among the differentially expressed transcripts (between the coral- and macroalgal-DOM treatments) were enriched compared to the metazoan and the prokaryotic GO-annotated transcripts in the reference transcriptomes. The enriched metazoan and prokaryotic GO categories were obtained with the Fisher’s Exact Test (FDR < 0.05), using the metazoan and the prokaryotic annotated transcripts as two separate reference sets.

## Results

### Transcriptome assembly and annotation

The sequencing of 18 cDNA libraries yielded a total of 830,367,692 reads, which resulted in ~ 37 million reads per sample after trimming. Basic sequencing metrics, including raw and trimmed reads and quality of the transcriptomes, can be seen in Table S1. Percentage of GC was even through all our samples, between 53 and 55% in *P. angulospiculatus* and 48–52% in *H. vansoesti* and changed marginally with read cleaning (Table S1). Using SortMeRNA, we observed little to moderate ribosomal contamination among our sequences. Eukaryotic ribosomal content was relatively higher in *H. vansoesti* compared to *P. angulospiculatus* (14–22 and 8–14%, respectively), while the opposite was true for bacterial ribosomal content (4–6 and 9-15%, respectively). The original reads have been uploaded to the NCBI database under BioProject ID PRJNA772056.

For each sponge species separately, the reads from all nine samples (i.e., three replicates x three DOM treatments: coral-, macroalgal-, and diatom-DOM) were used to construct a reference transcriptome assembly (see Table 1 for statistics). A total of 577,453 transcripts and 344,473 ‘genes’ (i.e., Trinity components or ‘assembled genes’ as identified in the Trinity pipeline) were present in *P. angulospiculatus* and 390,371 transcripts and 157,695 ‘genes’ in *H. vansoesti* (Table 1).

**Table 1:** Statistics of the assembled reference transcriptomes of *Plakortis angulospiculatus* and *Haliclona vansoesti*. In brackets are given the percentages of the number of transcripts that received a certain annotation

Species	*P. angulospiculatus*	*H. vansoesti*
**Number of transcripts**	577,453	390,371
**Number of Trinity ‘genes’**	344,473	157,695
**Total bp in assembly**	744,143,727	335,442,941
**Max contig length**	136,032	60,113
**Mean contig length (bp)**	1289	859
**Median contig length (bp)**	482	409
**% GC**	49	39
**N20 contig length**	8276	4602
**N50 contig length**	3304	1651
**Number of contigs in N50**	55,934	47,248
**Number of transcripts over 1000 bp**	165,469	79,227
**Alignment rate to reference transcriptome**	88.98%	88.95%
**Transcripts w/blast hit (NR-Eukaryota)**	86,305 (15%)	94,220 (24%)
**Transcripts w/blast hit (NR-Prokaryota)**	142,360 (25%)	13,682 (4%)
**Transcripts w/blast hit (SP-Metazoa)**	170,324 (29%)	109,050 (28%)
**Transcripts w/blast hit (SP-Prokaryota)**	215,940 (37%)	46,627 (12%)
**Transcripts w/GO term (SP-Metazoa)**	124,471 (22%)	108,524 (28%)
**Transcripts w/GO term (SP-Prokaryota)**	210,685 (36%)	43,770 (11%)
**Proteins w/KEGG term**	39,657	25,883

*Abbreviations*: *NR Nr* database, *SP* Swiss-Prot database

To test the completeness of our transcriptomic datasets we used the BUSCO approach [35], which revealed our dataset to be more complete in *P. angulospiculatus* than in *H. vansoesti* for the prokaryotic (61 and 36%, respectively) set of genes, while both species had similarly high outputs for the metazoan (87 and 90%, respectively) and the eukaryotic set (93 and 92%, respectively), as can be seen in Table 2.

**Table 2:** Completeness of the transcriptomic datasets of *Haliclona vansoesti* and *Plakortis angulospiculatus* assessed with BUSCO v5

*Species*	Reference gene set	C	S	D	F	M
***P. angulospiculatus***	Eukaryota	92.5%	8.6%	83.9%	6.3%	1.2%
	Metazoa	87.1%	7.5%	79.6%	8.5%	4.4%
	Prokaryota	61.3%	15.3%	46.0%	8.1%	30.6%
***H. vansoesti***	Eukaryota	91.8%	9.4%	82.4%	6.7%	1.5%
	Metazoa	89.7%	11.2%	78.5%	6.3%	4.0%
	Prokaryota	36.3%	12.9%	23.4%	16.1%	47.6%

*Abbreviations*: *C* Complete, *S* Single copy, *D* Duplicate, *F* Fragmented, *M* Missing core genes

To annotate our data, we used BLAST, DIAMOND, Blast2GOPRO and KEGG platforms. Using the *nr* database (containing eukaryotic and prokaryotic genes), the hits obtained against eukaryotic genes for both sponge transcriptomes were very similar, but the HMA sponge *P. angulospiculatus* showed ten times more hits against the prokaryotic genes than the LMA sponge *H. vansoesti* (Table 1). We also performed KEGG annotation on our *de-novo* transcriptomes using the KAAS-KEGG automatic annotation server (Additional File 1). KEGG pathway recovery was good in both *P. angulospiculatus* and *H. vansoesti* with, respectively, 39,657 and 25,883 transcripts annotated to existing KEGG terms (metazoa and prokaryotes). The higher number of KEGG annotations in *P. angulospiculatus* compared to *H. vansoesti* was reflected in the completeness (i.e., when all genes involved in a pathways are expressed) of the modules for carbon metabolism, nitrogen metabolism and ABC transporters (Table S2). For example, archaeal pentose phosphate pathway, methane oxidation, assimilatory nitrate reduction, denitrification, complete nitrification and transporters of extracellular nitrate/nitrite (NRT) were fully expressed only in *P. angulospiculatus* (Table S2). However, the KEGG nitrogen fixation pathway was missing in both species. Among the eukaryotic-type ABC transporters—a ubiquitous superfamily of membrane proteins that is mainly responsible for transportation of substrates across membranes—, 18 were present in both sponge species, with an additional nine only in *P. angulospiculatus* and four only in *H. vansoesti* (Table S2). Among the prokaryotic type, some ABC transporters were annotated in both sponge species, but several others were complete only in one of the two species: maltose and glycerol transporters were annotated only in *H. vansoesti*, while in *P. angulospiculatus* complete annotation was retrieved for more transporters, including those transporting ions, monosaccharides, oligosaccharides, phospholipids, phosphate, amino acids, urea, peptides, and other substrates (Table S2).

### Differential expression and functional enrichment analyses of coral- versus macroalgal-DOM feeding

**Fig. 1:**
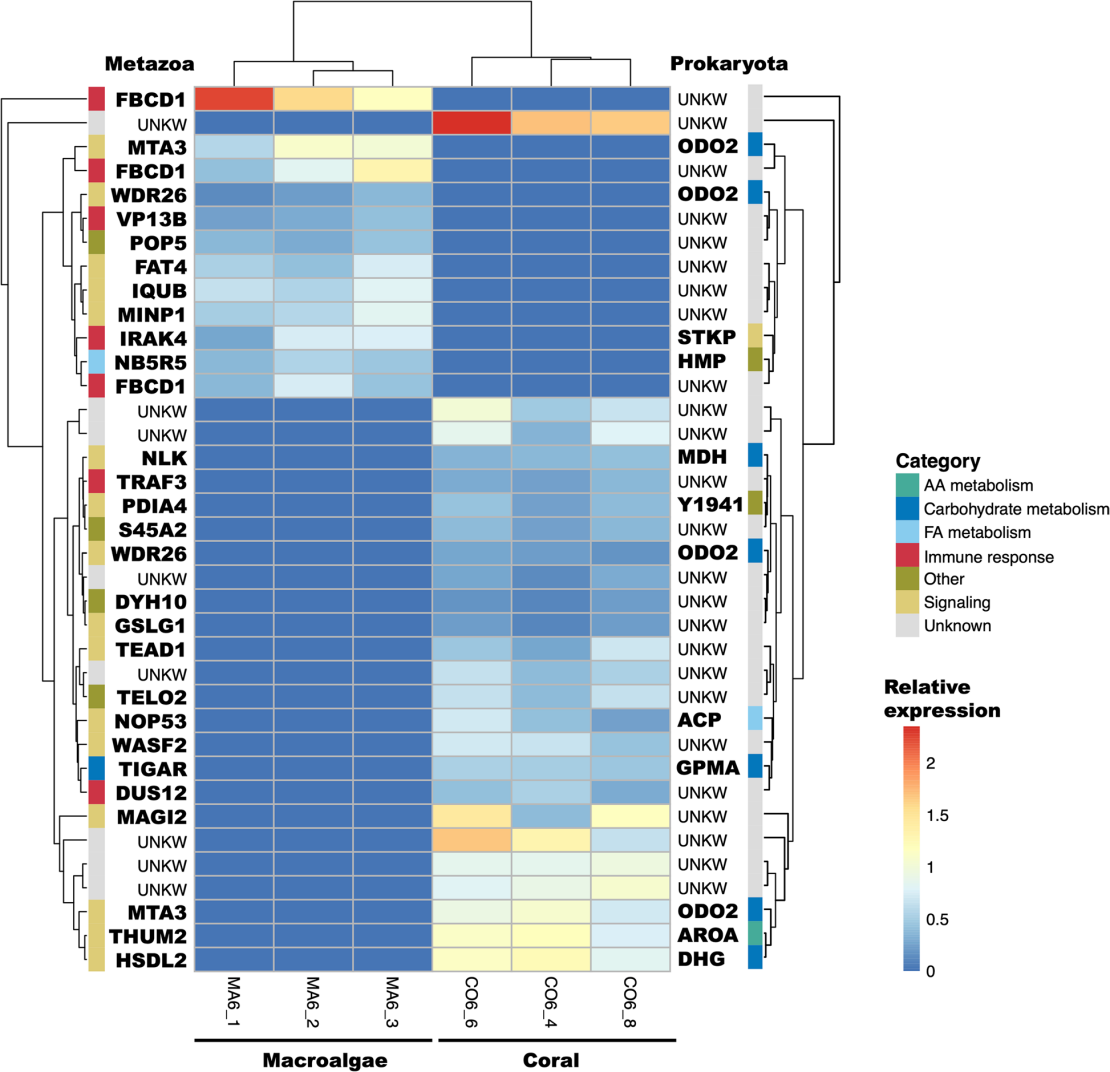
Heatmap depicting the expression level of the differentially expressed transcripts in individuals of the high microbial abundance species *Plakortis angulospiculatus.* On the left are listed the annotations obtained with the Swiss-Prot metazoan database and on the right are listed the annotations obtained with the Swiss-Prot prokaryotic database. The scale for relative expression values (log2 scaled) increases from blue to red. The functions of the annotated transcripts are grouped in categories and depicted by the different colors. AA, amino acids; FA, fatty acids; UNKW, unknown.

**Fig. 2:**
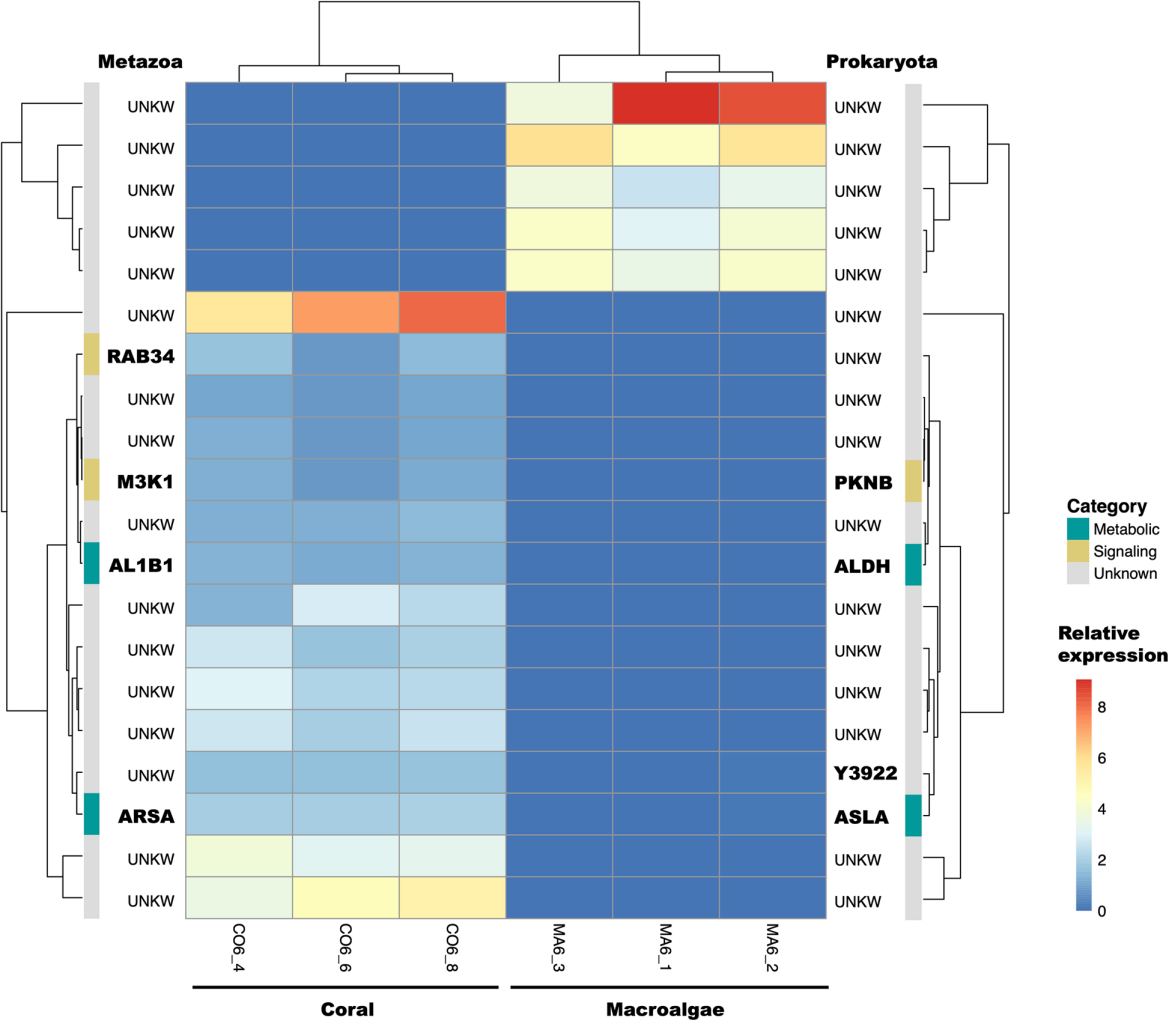
Heatmap depicting the expression level of the differentially expressed transcripts in individuals of the low microbial abundance species *Haliclona vansoesti.* On the left are listed the annotations obtained with the Swiss-Prot metazoan database and on the right are listed the annotations obtained with the Swiss-Prot prokaryotic database. The scale for relative expression values (log2 scaled) increases from blue to red. The functions of the annotated transcripts are grouped in categories and depicted by the different colors. AA, amino acids; FA, fatty acids; UNKW, unknown

To compare the differences in response to coral- versus macroalgal-DOM feeding in our two target sponges, we evaluated the differential expression of transcripts. Subsequently, a functional GO enrichment analysis was carried out on the differentially expressed transcripts between the coral- and macroalgae-DOM treatments. In both species, the transcriptional profile did not vary much among the DOM feeding treatments, but we consistently retrieved more differentially expressed transcripts in the coral-DOM treatment than in the macroalgae-DOM treatment (Figs. 1 and 2 and S1). In the HMA species *P. angulospiculatus*, 37 transcripts were differentially expressed between the coral- and macroalgal-DOM treatments, of which twenty-five were upregulated in the coral-DOM treatment and twelve in the macroalgal one (Fig. 1 and Additional File 2). Twenty-nine out of 37 transcripts were annotated with the Swiss-Prot metazoan and twelve with the Swiss-Prot prokaryotic databases (Figs. 1 and S2).

In the LMA species *H. vansoesti*, we found a total of 20 differentially expressed transcripts, of which fifteen were upregulated in the coral-DOM treatment and five upregulated in the macroalgae-DOM treatment (Fig. 2 and Additional File 2). Only four out of the twenty differentially expressed transcripts could be annotated using the Swiss-Prot metazoan database and four with the Swiss-Prot prokaryotic database (Figs. 2 and S2). The results of the differential gene expression and GO enrichment analysis are provided in full as Additional File 2. The sample correlation matrix and heatmap of relative expression for differentially expressed transcripts can be seen in Fig. S1. In *P. angulospiculatus* there was overlap in the clustering of the coral- and macroalgal-DOM treatments (Fig. S1A-B), while in *H. vansoesti*, the two treatments clustered separately from each other (Fig. S1C-D).

**Fig. 3:**
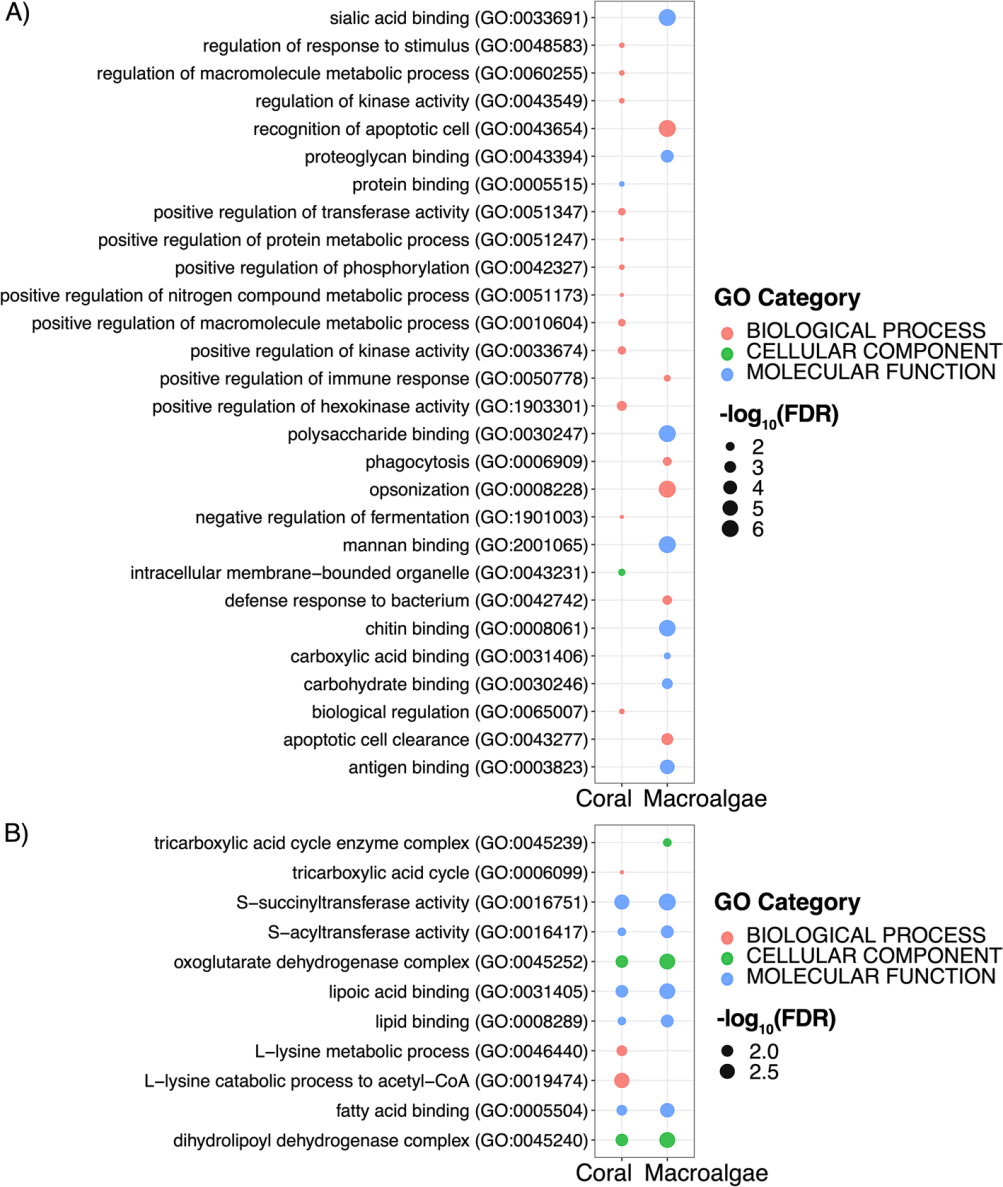
Plot of the representative enriched GO terms (FDR < 0.05) identified in *Plakortis angulospiculatus* in the coral- and macroalgal-DOM treatments based on A) the metazoan and B) the prokaryotic annotated transcripts. The size of the dot indicates the significance of the enrichment expressed as -log_10_(FDR), the colors represent the different GO categories

In *P. angulospiculatus*, there were several GO categories enriched among the differentially expressed transcripts between coral- versus macroalgal-DOM feeding (Fig. 3). In the metazoan set, there were a total of 74 GO terms enriched (Fig. S3). Among the GO terms enriched in the coral-DOM treatment (i.e., as compared to macroalgal-DOM) there was the ‘positive regulation of cellular metabolic processing’, including those involving nitrogen, phosphorus, kinase, and hexokinase activity and binding and transfer of proteins. At the same time, there was the ‘negative regulation of fermentation’, specifically of the ‘catabolic processing of glucose to lactate via pyruvate’ and of ‘NAD metabolic processes’ (Figs. 3A and S3). Three out of the 25 differentially expressed transcripts upregulated in the coral-DOM treatment, namely *Fructose-2,6-bisphosphatase (TIGAR), TNF receptor-associated factor 3 (TRAF3), and Transcriptional enhancer factor TEF-1 (TEAD1)*, were responsible for over 70% of the enriched GO terms (Additional File 2). In the macroalgal-DOM treatment (i.e., as compared to the coral-DOM treatment), the GO terms enriched in the metazoan gene set comprised the ‘positive regulation of immune response’, the ‘defense to Gram-positive and -negative bacteria’, ‘phagocytosis’, and the ‘recognition and clearance of apoptotic cells’, along with the ‘binding to acetylated compounds’, such as chitin, mannan, proteoglycan, and sialic acid (Figs. 3A and S3). These terms were all related to the upregulated transcript *Fibrinogen C domain-containing protein 1* (FBCD1; Additional File 2). In the prokaryotic transcripts of *P. angulospiculatus* there were a total of 28 GO terms enriched (Fig. S4). GO term expression was similar in both coral- and macroalgal-DOM treatments with enrichment in the TCA cycle metabolic processes through the ‘oxoglutarate dehydrogenase complex’ (i.e., oxoglutarate dehydrogenase, dihydrolipoyl dehydrogenase and succinyltransferase), and the ‘binding of lipids and fatty acids’ (Figs. 3B and S4). The GO term enrichment in both treatments was driven by the upregulation of two transcripts of the gene *Dihydrolipoyllysine-residue succinyltransferase component of 2-oxoglutarate dehydrogenase complex* (ODO2; Additional File 2). Because the majority of the differentially expressed transcripts were not annotated in *H. vansoesti*, we did not find significantly enriched GO categories in any of the treatments in this sponge species.

## Discussion

We compared the transcriptomic response of two sponge species—the high microbial abundance sponge *Plakortis angulospiculatus* and the low microbial abundance sponge *Haliclona vansoesti*—after feeding on coral- and macroalgal-DOM, the two main natural DOM sources available on coral reefs [45]. Our *de-novo* assembled reference transcriptomes showed that both sponge species expressed a wide metabolic repertoire, but that the higher abundance of associated microbes in *P. angulospiculatus* likely provided additional functions compared to *H. vansoesti*. For example, pathways related to the metabolism of nitrogen, methane, and substrate transporters were complete in *P. angulospiculatus*, but not in *H. vansoesti*. The gene expression varied significantly between the coral- and the macroalgal-DOM treatment in both sponge species, but the number of differentially expressed transcripts was relatively small, compared to other transcriptomic studies in sponges [46–50]. This indicates that the response to short-term feeding (6 h) on the two DOM sources was rather moderate. Furthermore, our interpretation of the functional response of *H. vansoesti* and *P. angulospiculatus* to DOM-feeding was limited by the transcriptome annotation. This is a pervasive problem in transcriptomic studies of non-model species like sponges [47, 51, 52], and is exacerbated by the absence of genomes for these sponge species. Especially for *H. vansoesti*, only less than 20% of the differentially expressed transcripts could be annotated based on public databases.

### Metazoan transcripts

**Fig. 4:**
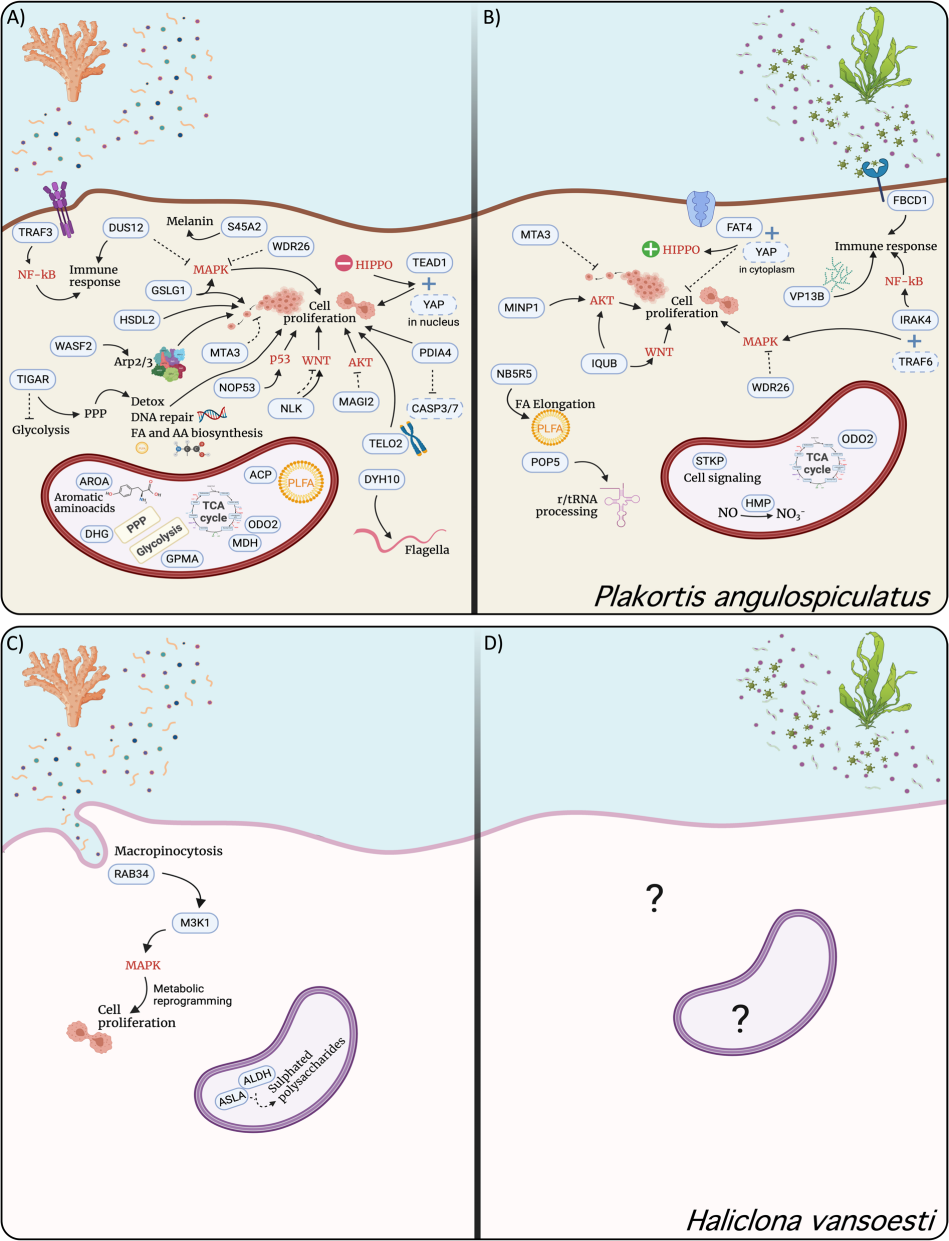
Suggested reconstruction of the response of *Plakortis angulospiculatus* (top panel) and *Haliclona vansoesti* (bottom panel) after exposure to coral (**A** and **C**) and macroalgae (**B** and **D**) dissolved organic matter. Schematic representation based on the set of annotated differentially expressed transcripts between the coral- and macroalgal-DOM treatments, which are circled by a full line. Transcripts circled by a dashed line are part of the non-differentially expressed set, but included here because their presence could regulate specific cellular functions when associated with some of the differentially expressed transcripts. Transcripts upregulated in the metazoan set are depicted in the background tissue, while transcripts upregulated in the prokaryotic sets are depicted inside the bacterium shape. Activation is indicated by full arrows, while inhibition by dashed lines. Signaling pathways are written in red color. Abbreviations: NF-kB, nuclear factor kappa-B; MAPK, mitogen-activated protein kinase; AKT, serine/threonine-protein kinase; Arp2/3, actin related protein 2/3 complex; PPP, pentose phosphate pathway; FA, fatty acids; AA, amino acids; TCA, tricarboxylic acid cycle. Created with BioRender.com

Feeding on coral- versus macroalgal-DOM elicited in both sponge species the expression of an array of transcripts involved in signaling pathways (Fig. 4), which can regulate cell growth, proliferation, differentiation, survival, apoptosis and immunity in metazoans [53].

#### Cell proliferation

The largest portion of the differentially expressed transcripts after coral- versus macroalgal-DOM feeding in the HMA species *P. angulospiculatus* were related to several signaling pathways (Figs. 1 and 4A and Additional File 2), including mitogen-activated protein kinase (MAPK), Wnt/β-catenin, serine/threonine-protein kinase (AKT), p53, and Hippo signaling, which are related to developmental processes but also heavily involved in tumor development and metastasis in humans and mice. All these pathways have also been detected in the genome of the sponge *Amphimedon queenslandica* [54] and transcriptomes of other sponges [50, 51, 55, 56]. Some sponge cells (especially those considered the stem cell complements: archaeocytes and choanocytes), share at least one characteristic with germ line cells and tumor cells of higher metazoan phyla; they contain high levels of telomerase activity, suggesting that they possess high proliferation and differentiation capacity [57–59]. Rapid cell proliferation has been widely reported in the filter-feeding cells (choanocytes) of sponges [60, 61] and as a mechanism of cell turnover and regeneration in sponges [62–66]. Interestingly, we also found a transcript involved in telomerase activity, i.e., *Telomere length regulation protein* (*TELO2*), upregulated in *P. angulospiculatus* after feeding on coral-DOM (Fig. 1). We thus suspect that the cellular signaling pathways usually upregulated in tumor cells are likely related to cell proliferation and differentiation in sponges, as previously described in other sponges [57]. However, experiments targeting molecular mechanisms of cell proliferation, such as gene knock down or silencing, in sponges are required to verify this.

Among the differentially expressed transcripts related to cell signaling in *P. angulospiculatus*, we found several that could positively regulate cell proliferation in coral- compared to macroalgal-DOM feeding. Transcripts such as *Protein disulfide-isomerase (PDIA4)*, *Ribosome biogenesis protein (NOP53)*, *Golgi apparatus protein 1 (GSLG1), Hydroxysteroid dehydrogenase-like protein 2 (HSDL2)*, and *Wiskott-Aldrich syndrome protein family member 2 (WASF2)* (Fig. 1) are indeed associated with tumor cell proliferation, growth and metastasis [67–71]. Furthermore, in the coral-DOM treatment as opposed to the macroalgal one, the upregulation of the gene *Membrane-associated guanylate kinase (MAGI2)* could regulate cell division by keeping cells from proliferating too rapidly or in an uncontrolled way by suppressing serine/threonine-protein kinase (AKT) activation [72]. Another interesting comparison between the coral- and macroalgal-DOM treatments in *P. angulospiculatus* was the differential expression of two transcripts involved in the Hippo signaling pathway. Cell proliferation could be enhanced through this pathway after feeding on coral-DOM as compared to macroalgal-DOM, since the gene *Transcriptional enhancer factor TEF-1 (TEAD1)* was upregulated in the coral- versus macroalgal-DOM treatment and vice versa for the gene *Protocadherin (FAT4).* In fact, when Hippo signaling is off, the Yes-associated protein (*YAP*) translocates to the nucleus where it can bind *TEAD1* with anti-apoptotic and pro-proliferative effects [73]. In contrast, when Hippo signaling is on, the gene *FAT4* can keep *YAP* in the cytoplasm, thereby downregulating cell proliferation [74, 75].

The link to cell proliferation processes, especially induced after feeding on coral-DOM, was also partially observed in the LMA sponge *H. vansoesti*. In *H. vansoesti*, the transcripts *Mitogen-activated kinase kinase kinase 1 (M3K1)* and *Ras-related Rab-34 (RAB34)* were upregulated in the coral-DOM treatment as compared to the macroalgal-DOM one (Figs. 2 and 4B and Additional File 2). *M3K1* is one of the furthest upstream kinases of the abovementioned MAPK signaling cascade. The *M3K1* module is activated by cell surface receptors, such as growth factors, G-protein-coupled receptors (GPCRs), small GTPases, and cellular stress [76]. The gene *RAB34* encodes a small GTPase involved in protein transport and therefore is a precursor of various MAPK pathways [77]. Furthermore, the gene *RAB34* has been shown to be involved in the regulation of macropinocytosis [78, 79]. Evidence for macro-pinocytic activity has been found in freshwater [80, 81] and marine sponges [82]. We, therefore, hypothesize that macropinocytosis is one of the candidate mechanisms for DOM uptake by the filter-feeding cells (choanocytes) of sponges [83–85] and this could in turn activate the MAPK pathways and stimulate metabolic reprogramming towards a number of cellular functions, including cell proliferation (Fig. 4).

Nutrient supply is an important factor regulating cell proliferation since it is an energetically costly process [86]. Starvation, for example, likely causes a reduction in cell proliferation in sponges [64, 87], therefore changes in diet composition can also play an important role in controlling cell growth, proliferation, and survival. For example, in *P. angulospiculatus* fed with coral-DOM compared to macroalgal-DOM we found upregulation of *TIGAR*, which is a modulator of glucose metabolism for energy production [88–90]. By suppressing glycolysis, *TIGAR* causes the accumulation of glucose 6-phosphate that is then diverted into the pentose phosphate (PP) pathway to generate nucleotides, NADPH, and antioxidants, which help repair DNA, reduce reactive oxygen species and support rapid cell proliferation [91, 92]. While macroalgae release DOM composed of labile carbohydrates, which can be shunted directly in the PP pathway [17], coral-DOM is usually richer in proteins and fatty acids [15, 93]. Therefore, the upregulation of a mechanism that activates the PP pathway may be required to assist rapid cell proliferation in sponges feeding on coral-DOM.

#### Immune response

Transcripts related to the immune response were upregulated in *P. angulospiculatus* when comparing coral- versus macroalgal-DOM feeding (Figs. 1 and 4 and Additional File 2). The innate immune response is evolutionarily conserved across many different taxa and can be triggered by the presence of cellular compounds of microbial pathogens, such as chitin, peptidoglycan, and lipopolysaccharides [94]. Even after (0.7 μm) filtration, small pathogens or their cellular components are likely present in naturally produced DOM sources, especially in the macroalgal-DOM, as it has been shown to induce the growth of copiotrophic, pathogen-like microorganisms [17]. Furthermore, macroalgae possess lipopolysaccharides that are mainly sulfated galactans, fucans or heteroglycuronans, which are known to induce inflammatory responses [95]. In our GO enrichment analyses we found that the immune response observed after macroalgal-DOM feeding was mostly driven by the gene *Fibrinogen C domain-containing protein 1 (FBCD1)*. *FBCD1* binds to acetylated structures such as chitin, N-acetylated carbohydrates, and amino acids [96, 97], and it has already been found to contribute to the gene repertoire for immune recognition in invertebrates [98] and sponges [48]. Along with *FBCD1*, the upregulated gene *Interleukin-1 receptor-associated kinase 4 (IRAK4)* after macroalgal-DOM feeding could have played a critical role in initiating innate immune response against foreign pathogens, because its overexpression activates nuclear factor-kappa B (NF-κB), which regulates the immune response to infections [99].

Immune functions were also induced after coral-DOM feeding by the transcripts *TNF receptor-associated factor 3 (TRAF3)* and *Dual specificity protein phosphatase 12 (DUS12)*. *TRAF3* plays important roles in mediating innate immune receptor and cytokine receptor signals [100] and belongs to the tumor necrosis factor receptor-associated factors (TRAFs), which have been found in the immune repertoire of sponges [48, 51, 52, 101]. *DUS12* also regulates immune responses by inhibition of various MAPK cascades and production of proinflammatory cytokines and chemokines in response to toll-like receptors (TLRs) activation and microbial infection [102, 103].

Among the transcript differentially expressed in *H. vansoesti*, we did not find any with an annotation related to the immune response. However, given the high number of transcripts with unknown functions found in *H. vansoesti,* we cannot exclude that some of these transcripts could be involved in some immune related functions yet to be described.

#### Other metabolic responses

In the comparison between the coral- and macroalgal-DOM treatments in *P. angulospiculatus* we also found differential expression of transcripts related to other metabolic functions than cell proliferation and immune responses. The transcripts *NADH-cytochrome b5 reductase-like (NB5R5)*, involved in fatty acid elongation [104], and *Ribonuclease P/MRP protein subunit (POP5)*, involved in the processing of precursor-rRNA and -tRNA [105], were upregulated after feeding on macroalgal- versus coral-DOM. On the other hand, the transcripts *axonemal dynein heavy chain 10 (DYH10)* and *membrane-associated transporter protein (S45A2)* were upregulated in the coral-DOM treatment. *DYH10* is a microtubule-associated motor protein [106] that could be related to the movement of flagella in choanocyte cells, while *S45A2* elevates the pH in melanocytes and promotes tyrosinase activity and melanin synthesis [107]. Melanin has antioxidant properties and is produced by sponge-associated bacteria [108], but it has been also found deposited in sponge tissues [109], and therefore it is yet uncertain if the host itself is also able to produce melanin. Our results of upregulation of melanin synthesis pathways by *P. angulospiculatus* could then be the first indication of host-driven melanin production at the molecular level.

### Prokaryotic transcripts

A major limitation in meta-transcriptomic analysis of non-model organisms is the annotation of the transcripts, especially of the prokaryotic ones. Given that we are working with *de-novo* assemblies without reference genomes, it is important to be aware of the difficulties in assigning certain transcripts to either the host or the associated microbiota, especially for certain housekeeping genes. In the LMA species *H. vansoesti* we found two transcripts with similar annotation in both the metazoan and prokaryotic sets, an *arylsulfatase (ASLA)* and an *aldehyde dehydrogenase (ALDH),* upregulated in the coral-DOM as compared to the macroalgal-DOM treatment (Fig. 2). Whether these transcripts are of prokaryotic or eukaryotic origin is not certain, but our competitive annotation against the complete *nr* database suggests that the origin of these transcripts could be prokaryotic. Furthermore, these transcripts have previously been found to form a cluster in a sponge associated microbiome [110]. The combination of arylsulfatase with dehydrogenase and ABC transporters may be an important group involved in the utilization of sulphated polysaccharides by the sponge microbiota [110].

Annotation of prokaryotic transcripts was more successful in the HMA species *P. angulospiculatus*, given the higher abundance of microbes found within the sponge tissue. Among the differentially expressed transcripts with prokaryotic annotation in *P. angulospiculatus,* we found that two different transcripts of the gene *ODO2* were upregulated in both the coral- and macroalgal-DOM treatment. As shown by the GO enrichment analysis, this gene is known for its role in the TCA cycle and its upregulation confirms the relevance of this metabolic pathway for energy production in sponge associate microbes. The upregulation of the same gene in both feeding treatments could point to a different microbe expressing it, therefore, a community shift associated with the different DOM feeding treatments cannot be excluded. Additionally, other transcripts related to carbohydrate degradation pathways were upregulated in the coral- compared to the macroalgal-DOM treatment, including the gene encoding for *malate dehydrogenase (MDH)*, another enzyme that takes part in the TCA cycle, *glucose 1-dehydrogenase (DHG)*, which is involved in the PP pathway, and a *phosphoglycerate mutase (GPMA)* that contributes to glycolysis/gluconeogenesis. This corroborates our previous finding in which we identified bacterial groups with a predicted genomic repertoire for TCA cycle, glycolysis and PP pathway to be active DOM incorporators in *P. angulospiculatus* [29]. Furthermore, in the coral-DOM treatment we found activation of fatty-acid biosynthesis through expression of *acyl carrier protein (ACP)* [111], and of aromatic amino acids biosynthesis, through expression of *3-phosphoshikimate 1-carboxyvinyltransferase (AROA)* [112].

The exposure to macroalgal-DOM instead stimulated two other prokaryotic transcripts, namely *Flavohemoprotein (HMP)* and *Serine/threonine-protein kinase (STKP)*. The upregulation of these two transcripts suggest that the sponge microbiota may experience and activate mechanisms to counteract higher levels of nitrosative and/or oxidative stress produced by the host immune system when the holobiont is exposed to pathogens present in the macroalgal-DOM [113]. *HMP* is a flavohemoprotein that is activated to counteract nitrosative stress by oxidation of NO to NO_3_^−^ under aerobic or microaerobic conditions [114], while *STKP* is a protein kinase involved in signal transduction pathways that regulate various cellular processes, including positive regulation of cell wall metabolism, pyrimidine biosynthesis, DNA repair, iron uptake, oxidative stress, and negative regulation of competence [115, 116].

## Conclusions

We conclude that the sponge host is expressing molecular responses that involve immune response and signal reception and transduction through several signaling cascades regardless of the nature of the DOM upon which the sponges were feeding (Fig. 4). Cell proliferation is likely an important mechanism in the studied sponges and it seems to be positively regulated after exposure to coral-DOM, but less so upon exposure to macroalgal-DOM. Sponges feeding on macroalgal-DOM in fact triggered a more ambiguous transcriptional response with regards to cell proliferation, inducing both positive and negative regulatory pathways, along with a stronger immune response. The sponge microbiota showed an active carbohydrate metabolism when exposed to both DOM sources, but it seems that whereas coral-DOM also induced lipid and amino acids biosynthesis, macroalgal-DOM caused a stress response. The differences in gene expression outlined in the comparison between the two DOM treatments may be driven by differences in organic macronutrients present in the two DOM sources and by the presence of small pathogens or bacterial virulence factors in the macroalgal-DOM source, which in turn affected the metabolism of the host and its associated microbiota. We are nonetheless limited in the interpretation of our results due to the large number of non-annotated transcripts in our analysis, especially for the sponge with low numbers of associated microbes. These transcripts could also be sponge-specific transcripts with a particular function in sponge metabolism yet to be discovered. Furthermore, the small number of differentially expressed transcripts between the coral- and the macroalgal-DOM treatment suggests that sponges could need either a shorter or a longer time to respond and adjust their metabolism. Longer-term experiments to evaluate the metabolic and physiological responses to both DOM sources should be performed in order to understand if the ecological function of sponges will be affected when reefs shift from coral towards algal dominance.

## Supplementary Information

**Additional file 1.**

**Additional file 2.**

**Additional file 3.**

## Data Availability

The original sequence reads have been uploaded to the NCBI database under BioProject ID PRJNA772056. All data analysed in this study are included in this published article and its additional information files.
