# Supplementary material for: Meta-transcriptomic comparison of two sponge holobionts feeding on coral- and macroalgal-dissolved organic matter
Source: BMC Genomics. 2022 Sep 29;23:674. doi: 10.1186/s12864-022-08893-y (PMC9520939; doi:10.1186/s12864-022-08893-y)
Supplement: Supplementary file 3 — Additional file 3. [file 12864_2022_8893_MOESM3_ESM.pdf]

Meta-transcriptomic comparison of two sponge holobionts feeding on coral- and macroalgal-dissolved organic matter

Sara Campana, Ana Riesgo, Evelien Jongepier, Janina Fuss, Gerard Muyzer, Jasper M. de Goeij

Supplementary figures

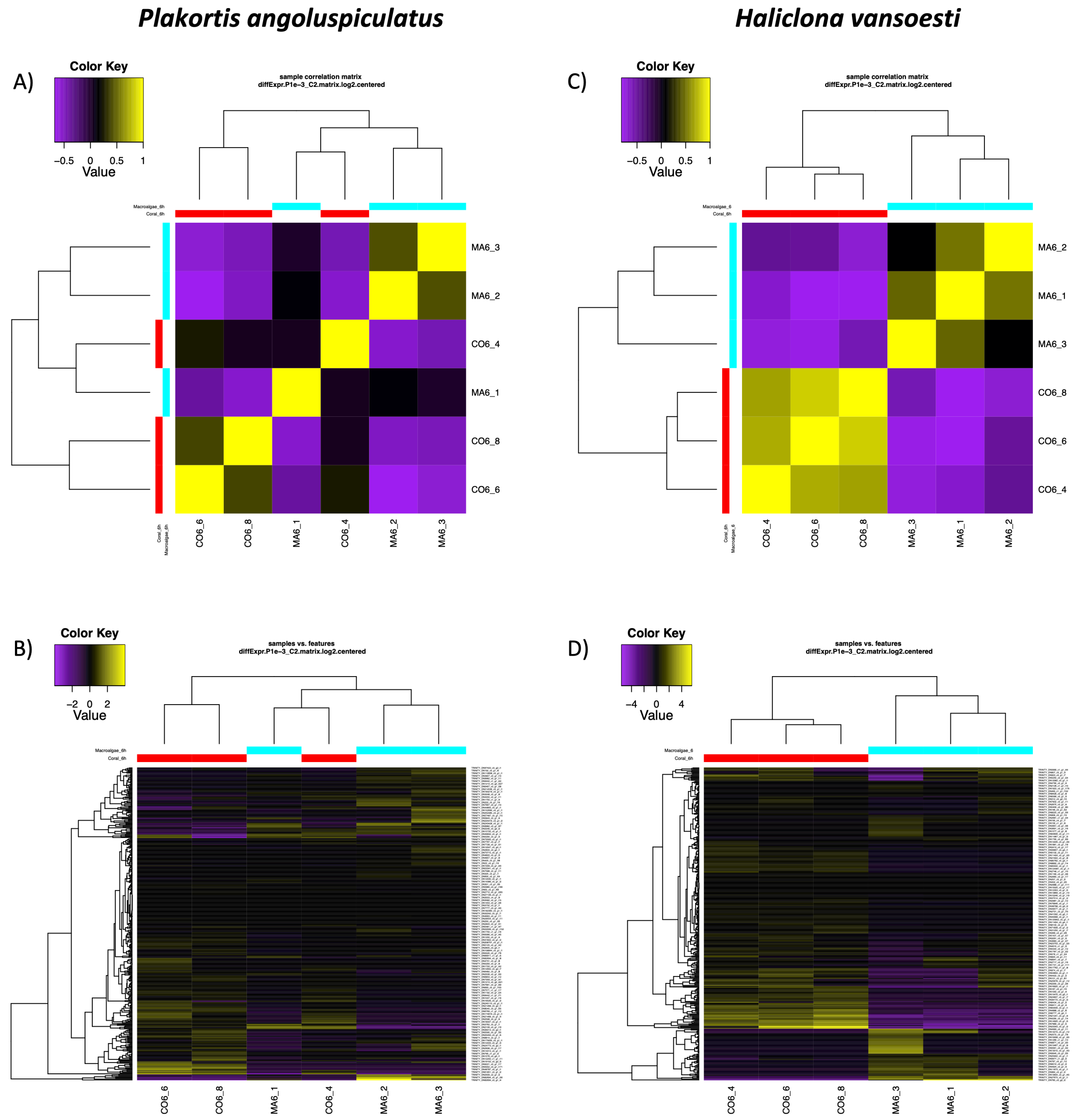

Figure S1: Differential expression results for *Plakortis angulospiculatus* (left) and *Haliclona vansoesti* (right). A) and C) Sample correlation matrices and B) and D) heatmaps of the differentially expressed transcripts across all the samples in the coral- and macroalgal-DOM treatments. Relative expression level increases from purple to yellow, the areas in black hues indicate few measurable differences between samples. The dendrogram represent the grouping of samples of the coral (in red color) and macroalgal (in light blue color) DOM treatments.

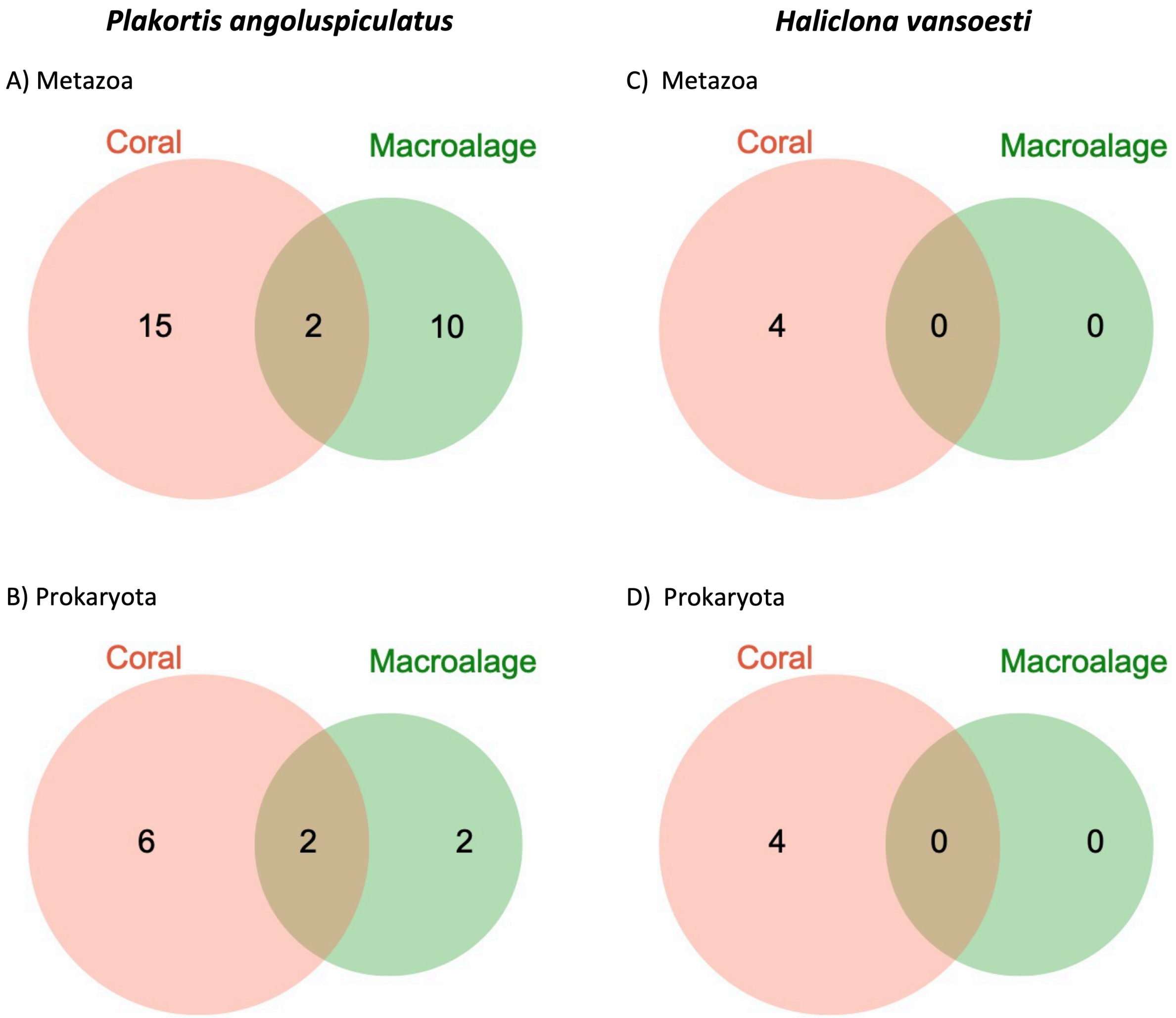

Figure S2: Venn diagrams of the unique and shared annotated transcripts differentially expressed between the coral- and macroalgal-DOM treatments in *Plakortis angulospiculatus* (left) and *Haliclona vansoesti* (right). A) and C) transcripts annotated with the Swiss-Prot metazoan database and B) and D) transcripts annotated with the Swiss-Prot prokaryotic database.

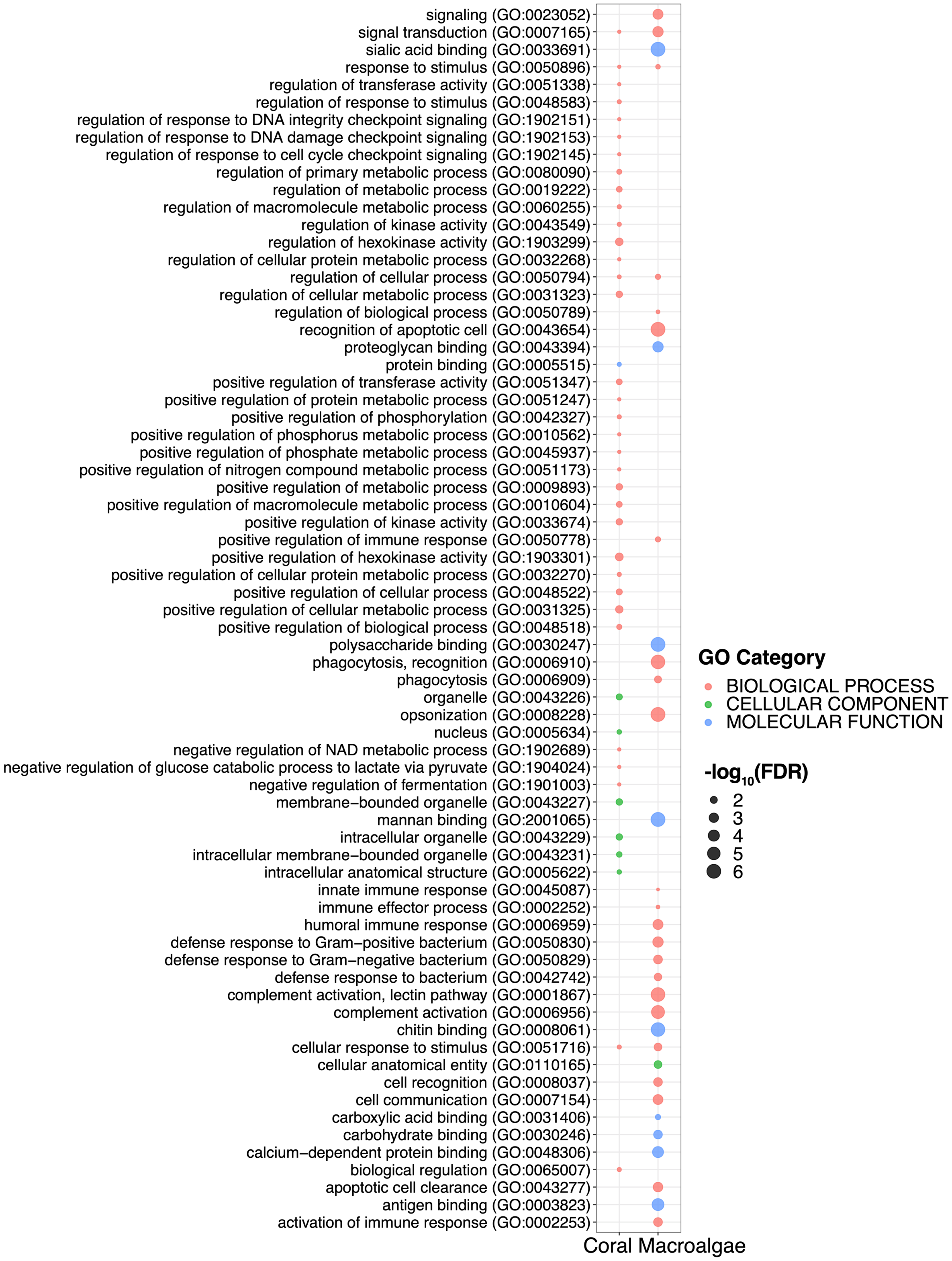

Figure S3: Plot of all enriched GO terms (FDR < 0.05) identified in in the metazoan transcript set of *Plakortis angulospiculatus* among the coral- and macroalgal-DOM treatments. The size of the dot indicates the significance of the enrichment expressed as -$\log_{10}$(FDR), the colors represent the different GO categories.

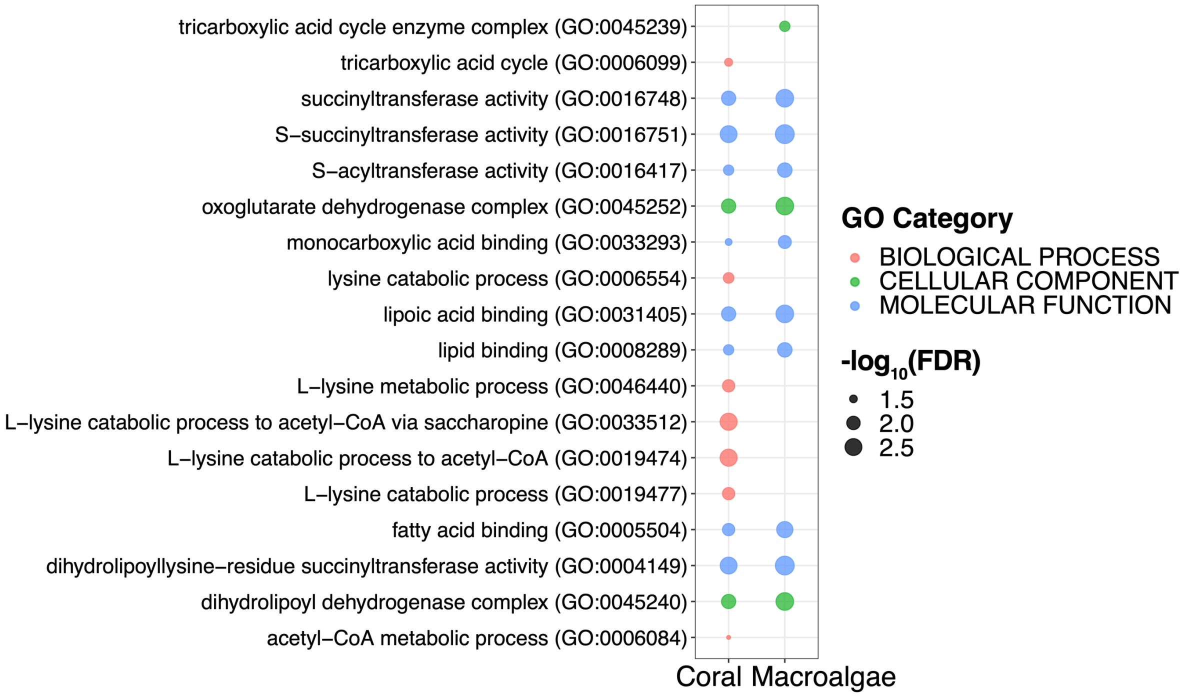

Figure S4: Plot of all enriched GO terms (FDR < 0.05) identified in in the prokaryotic transcript set of *Plakortis angulospiculatus* among the coral- and macroalgal-DOM treatments. The size of the dot indicates the significance of the enrichment expressed as -$\log_{10}$(FDR), the colors represent the different GO categories.

Supplementary tables

Table S1: Metrics for raw and trimmed read data.

Species	*DOM Treatment*	*Sample*	*N raw reads*	*%GC*	*N trimmed reads*	*%GC*	*Retained reads %*
Plakortis angulospiculatus	Coral	CO6_4	61,326,976	54	45,255,841	54	74
		CO6_6	50,064,685	54	36,592,071	54	73
		CO6_8	42,641,865	54	31,598,926	55	74
	Macroalgae	MA6_1	49,218,631	54	36,654,564	55	74
		MA6_2	58,893,425	54	43,794,140	54	74
		MA6_3	37,583,458	54	27,491,754	54	73
	Diatom	DI3_10	47,902,716	53	34,834,866	53	73
		DI3_11	48,473,538	54	35,232,215	54	73
		DI3_12	45,855,744	53	33,571,972	53	73
Haliclona vansoesti	Coral	CO6_4	48,225,848	48	35,266,862	49	73
		CO6_6	43,641,746	48	31,333,143	48	72
		CO6_8	42,050,768	50	29,998,238	50	71
	Macroalgae	MA6_1	49,284,127	51	35,014,528	51	71
		MA6_2	34,830,157	50	24,484,197	50	70
		MA6_3	49,166,280	50	34,601,090	50	70
	Diatom	DI3_10	37,601,350	52	26,817,836	52	71
		DI3_11	37,809,076	49	26,679,677	49	71
		DI3_12	45,797,302	52	32,239,001	52	70

Table S2: Complete KEGG pathways of interest.

KEGG category	*Pathway/Module*	*Plakortis angulospiculatus*	*Haliclona vansoesti*
Carbon metabolism	glycolysis (M00001)	✓	✓
	pentose phosphate pathway (M00004)	✓	✓
	citrate cycle (M00009)	✓	✓
	archaeal pentose phosphate pathway (M00580)	✓	✗
Methane metabolism	methane oxidation (M00174)	✓	✗
	formaldehyde assimilation (M00345)	✓	✗
Nitrogen metabolism	dissimilatory nitrate reduction (M00530)	✓	✓
	assimilatory nitrate reduction (M00531)	✓	✗
	denitrification (M00529)	✓	✗
	complete nitrification (M00804)	✓	✗
	nitrogen fixation (M00175)	✗	✗
	extracellular nitrate/nitrite (NRT) transporters	✓	✗
Eukaryotic-type ABC transporters	ABCA1, ABCA3, ABCB1, ABCB6, ABCB7, ABCB8, IrtA/B, MsbA, MdlA/B, VcaM, ABCC1, ABCC3, ABCC10, ABCD2, ABCD3, ABCD4, ABCG2, YddA	✓	✓
	PatA/B, ABCC4, CydC/D, PDR5	✗	✓
	ABCA2, ABCA5, ABCB9, HlyB, RaxB, Atm1, EfrA/B, LapB, HasD	✓	✗
Prokaryotic-type ABC transporters	iron, zinc, manganese, nucleoside, phosphate, L-amino acids, branched-chain amino acids, glutathione, sodium	✓	✓
	maltose/maltodextrine	✗	✓
	glycerol	✗	✓
	ions (taurine, alkanesulfonate, molybdate, spermidine/putrescine, glycine betaine/proline, osmoprotectants)	✓	✗
	monosaccharides (glucose/mannose, ribose, D-xylose, fructose, glycerol 3-phosphate)	✓	✗
	oligosaccharides (galactose/maltooligosaccharide, raffinose/stahyose/melibiose, sorbitol/mannitol, α-glucoside, threhalose/maltose, arabinooligosaccharide)	✓	✗
	phospholipids	✓	✗
	phosphate (phosphate, phosphonate)	✓	✗
	amino acids (arginine, lysine, histidine, glutamine, and neutral amino acids)	✓	✗
	urea	✓	✗
	peptides	✓	✗
	other substrates (including microcin-C, cobalt, biotin, lipo-poly/oligosaccharides, lipoproteins)	✓	✗

Abbreviations: ✓ , presence; ✗, absence
